# Diversity, Pathogenicity, and Current Occurrence of Bacterial Wilt Bacterium *Ralstonia solanacearum* in Peru

**DOI:** 10.3389/fpls.2017.01221

**Published:** 2017-07-18

**Authors:** Liliam Gutarra, Juan Herrera, Elizabeth Fernandez, Jan Kreuze, Hannele Lindqvist-Kreuze

**Affiliations:** ^1^Crop and Systems Sciences Division, International Potato Center Lima, Peru; ^2^Genetics, Genomic and Crop Improvement Division, International Potato Center Lima, Peru

**Keywords:** bacterial wilt, brown rot, potato, *Ralstonia solanacearum*, phylotype, sequevar, pathogenicity

## Abstract

The current bacterial wilt infestation level in the potato fields in the Peruvian Andes was investigated by collecting stem samples from wilted plants and detecting *Ralstonia solanacearum*. In total 39 farmers’ fields located in the central and northern Peru between the altitudes 2111 and 3742 m above sea level were sampled. *R. solanacearum* was detected in 19 fields, and in 153 out of the 358 samples analyzed. Phylogenetic analysis using the partial sequence of the endoglucanase gene on strains collected in Peru between 1966 and 2016 from potato, pepper, tomato, plantain or soil, divided the strains in phylotypes I, IIA, and IIB. The Phylotype IIB isolates formed seven sequevar groups including the previously identified sequevars 1, 2, 3, 4, and 25. In addition to this, three new sequevars of phylotype IIB were identified. Phylotype IIA isolates from Peru clustered together with reference strains previously assigned to sequevars 5, 39, 41, and 50, and additionally one new sequevar was identified. The Phylotype I strain was similar to the sequevar 18. Most of the Peruvian *R. solanacearum* isolates were IIB-1 strains. In the old collection sampled between 1966 and 2013, 72% were IIB-1 and in the new collection at 2016 no other strains were found. The pathogenicity of 25 isolates representing the IIA and IIB sequevar groups was tested on potato, tomato, eggplant and tobacco. All were highly aggressive on potato, but differed in pathogenicity on the other hosts, especially on tobacco. All IIA strains caused latent infection on tobacco and some strains also caused wilting, while IIB strains caused only few latent infections on this species. In conclusion, high molecular diversity was found among the *R. solanacearum* strains in Peru. Most of the variability was found in areas that are no longer used for potato cultivation and thus these strains do not pose a real threat for potato production in the country. Compared to the previous data from the 1990s, the incidence of bacterial wilt has decreased in Peru. The epidemics are likely caused by infected seed tubers carrying the clonal brown rot strain IIB-1.

## Introduction

Potato is an important food security crop with great potential for poverty alleviation and combating malnutrition in the developing world (FAO, 2009; Devaux et al., 2014). However, significant yield gaps have been observed in the small-holder’s fields caused by several constraints such as poor seed quality, soil fertility and plant pathogens (Gildemacher et al., 2011). One of the most notorious diseases affecting potato production in the developing world is bacterial wilt, caused by *Ralstonia solanacearum*. It is part of a diverse species complex (Fegan and Prior, 2005) affecting many plant species (Kelman, 1953; Hayward, 1994), including potato, tobacco, tomato, banana, peanut, and ginger (Elphinstone, 2005). The bacterium is difficult to control because it survives in water and in residues of infected plants in the soil. (Graham et al., 1979; Pradhanang et al., 2000; Caruso et al., 2005). Distribution of asymptomatic infected plant material has resulted in establishment of this pathogen in numerous ecoregions around the world (Hayward, 1991).

Recently, based on the combination of genomic and proteomic methods the taxonomy of the *R. solanacearum* species complex was revised to consist of three species: *R. pseudosolanacearum, R. solanacearum*, and *R. syzygii* (Safni et al., 2014; Prior et al., 2016). The species division is congruent with the previous Phylotype classification based on the sequence analysis of the ITS region and further subdivision by the DNA sequence of the endoglucanase gene (*egl*) (Fegan and Prior, 2005) so that the Phylotype II strains belong to the species *R. solanacearum*, Phylotype IV strains to *R. syzygii* and Phylotypes I and III to *R. pseudosolanacearum*. Before the use of DNA based analysis *R. solanacearum* was subdivided into five races based on host range (Buddenhagen et al., 1962; Pegg and Moffett, 1971; He et al., 1983; Hayward, 1991) and to five biovars, based on the capacity to use disaccharides or oxidize several hexose alcohols (Hayward, 1964; He et al., 1983; Hayward et al., 1989; French et al., 1995). At present the most common characterization method for *Ralstonia* populations involves partial sequencing of the *egl* gene coding for the Endoglucanase enzyme and assigning the strains into sequevars based on phylogenetic analysis (Fegan and Prior, 2005). Endoglucanase has an important role in pathogenicity of *Ralstonia solanacearum* being necessary for efficient invasion of plant roots and colonization of stems (Roberts et al., 1988; Denny et al., 1990).

Potato bacterial wilt is caused mostly by race 3/biovar 2A, which is now classified as phylotype IIB/ sequevar 1 (IIB-1) (Janse, 1996; Perez et al., 2008; Cellier and Prior, 2010; Siri et al., 2011). which probably originated from the Brazilian Amazon basin and can have co-evolved with many plant species (Wicker et al., 2012; Santiago et al., 2016). This potato infecting taxon has spread world-wide and the reference sequences from all over the world are highly homologous suggesting clonal propagation and a relatively recent international dissemination. In addition to the dominating IIB-1 strain, also other phylogenetically distinct strains have been found infecting potato, such as IIB-3, IIB-25, IIA-7 and IIA-35 (Cellier and Prior, 2010; Hong et al., 2012; Santiago et al., 2016).

In Peru, bacterial wilt was first recognized in banana and plantain (Revilla, 1967) and in tomato along the Amazon river (French and Sequeira, 1968, 1970). In potato, it was first discovered in the highlands of Ancash, Cajamarca, and La Libertad (Herrera and French, 1970) and in central Peru in San Ramon (Torres et al., 1985). Surprisingly it was also found in potato planted on virgin soils in the Amazon basin in the Northern part of the country in Yurimaguas suggesting that it was endemic to the area (Martin et al., 1981). Peruvian *R. solanacearum* isolates have been extensively characterized for phenotypic markers, such as biovar, virulence and pathogenicity using several hosts. These studies identified that the biovars 1, 2 and 3 exist in Peru and all can infect potato, however, biovar 3 is less pathogenic on potato (Martin et al., 1981). Biovar 2 was further divided in two different phenotypes: 2T (first known as phenotype A) and 2A (phenotype B), which had different virulence patterns and host ranges (French et al., 1993; Marin and El-Nashar, 1993). In addition, biovar 2A is much more aggressive than biovars 1 and 2T on potato (Gutarra et al., 2014). Thus, it has been known that the *R. solanacearum* population infecting potato in Peru is variable, but a detailed molecular characterization of the isolates has not been done. Similarly, the current infestation level of the potato fields in the main productions areas is unknown.

The objective of this study was to analyze the occurrence, genetic diversity and pathogenicity of *R. solanacearum* bacterium population that currently affects farmers’ fields in Peru and compare that to the diversity found in the old collection dating from 1960 to 2013. In this paper, we characterize the phylotype, endoglucanase (*egl)* sequevar and biovar of over 200 isolates to identify patterns of geographical distribution and pathogenicity on potato, tomato, eggplant and tobacco.

## Materials and Methods

### Sample Collection

The sampling locations at 2016 were selected based on the previous reports on the presence of potato brown rot in the area and consulting the local farmers and local phytosanitary officials. The samples were collected at the beginning of the potato flowering period, when tuber symptoms are rarely observed, thus we focused on collecting stem samples. Plants with wilting symptoms were uprooted in the field and a fragment of one principal stem cut at the stem base (ca. 10 cm above the ground) was collected from each plant. The scissors were disinfected between the cuttings of each sample by dipping in a 2% solution of NaOCl for 10 s. The stem piece of each sample was wrapped in towel paper and put inside a paper bag. In the laboratory, the stem pieces were washed individually with tap water and then allowed to dry on clean paper towel. From each of the 10-cm piece, a section of approximately 5 cm in length (eliminating 2–3 cm at each side) was cut using horticultural scissors. The scissors were flame sterilized between each sample. Each sample was dipped in a solution of 0.5% NaOCl contained in a plastic bag for 1 min; this solution was then discarded and a stem section was rinsed in the same plastic bag first with sterile distilled water, then with 96% alcohol, and finally with sterile distilled water (at least twice). From each of the 5-cm stem pieces cut previously, a 1–2-cm long fragment was cut using horticultural scissors. The stem fragments obtained from each sample was again placed in a new plastic bag and crushed with a hammer. Then 3 ml of sterile citrate extraction buffer (0.1 M citric acid, 0.1 M sodium citrate, pH 5.6) was added and the sample homogenized.

### Isolation and Maintenance of *Ralstonia solanacearum* Collections

For isolation of bacteria 20 μl of stem extract was plated on modified Kelman’s medium (MKM) (French et al., 1995), diluted by streaking and plates were incubated at 30°C for 2 days. Single colonies matching the description of that of *R. solanacearum* (irregular shape, fluidal, and entirely white or with a pink center) were selected and sub cultured on MKM without TZC (2, 3, 5 triphenyl tetrazolium chloride) at 30°C for two days. The bacteria colonies were transferred in screw cap test tubes containing 10 ml sterile water and maintained at 15°C. To verify that the cultures were pathogenic, all strains were inoculated in susceptible potato plants of the variety Desiree by injecting 20 μl of bacterial cell suspension (10^8^ cells ml^-1^) into the axil of the third leaf from the apical meristem and re-isolating the bacteria within a week from a wilted plant on MKM.

### Pathogenicity Tests on Tomato, Potato, Eggplant, and Tobacco

The pathogenicity test was performed using tomato (Rio Grande), potato (Canchan INIA), eggplant (Vivian) and tobacco (Samsun) plants. Seeds of tobacco, tomato and eggplant, and potato tubers were sown in plastic trays (30 cm × 40 cm) containing Promix Bx substrate. Approximately two weeks later when the plants were 10-15 cm long and had adequate roots, they were transplanted in 3.5 inch-diameter pots. 50 ml solution of fertilizer (N–P–K: 6-7-22) was applied at the time of planting. Plants were irrigated daily, except for 1 day prior to inoculation.

Bacterial suspensions were prepared by culturing strains for 48-h. at 30°C on MKM without TZC. The cells were harvested in sterile distilled water and the bacterial suspensions were measured using a spectrophotometer to determine concentrations.

For inoculation, 2 weeks after transplanting, the plant roots were damaged with a scalpel and immediately 40 ml of the bacterial suspension was poured into the soil (the final concentration of each isolate was of 5 × 10^7^ cells/g soil). A set of six plants was inoculated with each strain and placed in separated trays to prevent cross contamination. Plants were maintained until one month after inoculation, during which temperatures ranged from 16.9 to 28.5°C. The wilt incidence was recorded twice a week after the first wilting symptoms began to appear. Stems of those plants exhibiting no wilt were also analyzed, to detect latent infections by isolating the bacteria on MKM (French et al., 1995).

### DNA Extraction and Identification of Strains

For DNA extraction, the bacteria were streaked on MKM without TZC and the plates incubated at 30°C for 2 days. Then one colony was suspended in 100 μl of sterile NFW, boiled for 10 min and kept at -20°C prior to use.

The taxonomic identity of *R. solanacearum* was verified with primers *759/760* (Opina et al., 1997). PCR amplification was performed in a total volume of 15 μl containing 1X of PCR Buffer, 2.5 mM MgCl2, 0.2 μM each dNTP, 0.2 μM of each primer, 0.3 U of GoTaqG2 Flexi DNA polymerase (PROMEGA) and 1 μl DNA template. Amplification was performed in an Applied Biosystem Veriti thermocycler as follows: an initial denaturation step at 94°C for 2 min, followed by 30 cycles of denaturation at 94°C for 30 s, annealing at 59°C for 30 s, extension at 72°C for 17 s, and a final extension step at 72°C for 5 min. PCR products (10 μl) were analyzed by electrophoresis through 1% (w/v) agarose gels with 0.01 μl /ml GelRed^TM^ 10,000X (Biotium) and photographed under UV light in a The Chemidoc^TM^ MP Photodocumentation System (BIO-RAD). Fragments were compared with a 1 Kb Plus marker ladder. A positive identification was based on the presence of a 282 bp amplicon.

### Biovar Determination

Biovars of the strains were determined following the protocol described by Hayward (1964) and Hayward et al. (1989) using the basal medium (NH4H2PO4 1.0 g, KCl 0.2 g, MgSO4.7H2O 0.2 g, Difco bacto peptone 1.0 g, Agar 3.0 g, and Bromothymol blue 0.03 g per liter) containing 1% of each type of sugar (lactose, maltose, mannitol and sorbitol, D (+) trehalose or 1% of L (+) tartrate). An inoculum suspension (O.D. + 0.05 at 600 nm) of each strain was prepared in sterile distilled water from 2-day-old cultures. For the determination of biovars 40 μl of bacterial suspension was added in the microtiter plate containing 170 μl of the basal medium and for the phenotypes differentiation (2A and 2T) 200 μl of the suspension was added to 3 ml the basal medium without agar contained in test tubes. The sealed tubes and plates were incubated at 30°C for 6 days and the color reaction was visually evaluated.

### Phylotype Identification

Phylotype was determined by multiplex PCR using a set of phylotype-specific primers Nmult:21:1F, Nmult:21:2F, Nmult:22:InF, Nmult:23:AF, and Nmult21:RR (Fegan and Prior, 2005). Amplification was carried out in a total volume of 15 μl containing 1 X PCR buffer, 2.5 mM MgCl2, 0.2 mM of each dNTP, 0.2 μM of each primer, 0.3 U of Go*Taq G2 Flexi* DNA polymerase (PROMEGA) and 1 μl DNA template. Amplifications were performed in an Applied Biosystem Veriti thermocycler as follows: an initial denaturation step at 94°C for 2 min, followed by 30 cycles of denaturation at 94°C for 30 s, annealing at 59°C for 30 s, extension at 72°C for 23 s, and a final extension step at 72°C for 5 min. PCR products (10 μl) were analyzed by electrophoresis through 1% (w/v) agarose gels with 0.01 μl /ml GelRed^TM^ 10,000X (Biotium) and photographed under UV light in The Chemidoc^TM^ MP Photodocumentation System (BIO-RAD). DNA template from strains CIP-277 (phylotype I), CIP-435 (phylotype II), and CIP-358 (phylotype III) were used as positive amplification controls. The size of the amplified fragments was estimated by comparison with a 1 Kb Plus marker ladder.

### Sequevar Identification

PCR amplification of a 750-bp region of the *egl* gene was done using the primer pair Endo-F (5-ATGCATGCCGCTGGTCGCCGC-3) and Endo-R (5-GCGTTGCCCGGCACGAACACC-3) (Fegan et al., 1998; Fegan and Prior, 2005). Amplification was carried out in a total volume of 75 μl containing PCR buffer, 1.25 mM MgCl2, and 0.2 mM of each dNTP, 0.2 μM of each primer, 1.5 U of Go*Taq G2 Flexi* DNA polymerase (PROMEGA) and 3 μl DNA template. PCR was performed using an Applied Biosystem Veriti thermocycler. Amplifications were performed with an initial denaturation step at 95°C for 2 min, followed by 28 cycles of denaturation at 95°C for 30 s, annealing at 70°C for 30 s, extension at 72°C for 50 s, and a final extension step at 72°C for 5 min. PCR products (5 μl) were checked by electrophoresis through 1% (w/v) agarose gels with 0.01 μl /ml GelRed^TM^ 10,000X (Biotium) and photographed under UV light in The Chemidoc^TM^ MP Photodocumentation System (BIO-RAD). The size of the amplified fragments was estimated by comparison with a 1 Kb Plus marker ladder. Then PCR products were purified and sequenced by Macrogen services (Kumchun-ku, Seoul, Korea) using Endo-F and Endo-R primers. The raw sequences were assembled and aligned together with reference sequences of *R. solanacearum* strains obtained from NCBI sequence database. The DNA sequences of the isolates were assigned accession numbers MF461731 – MF461866 in the NCBI sequence database. The phylogenetic analysis was done based on partial sequences using MEGA7 (Kumar et al., 2016), phylogenetic trees were constructed from the genetic distance data by the Neighbor-Joining (NJ) and Maximum Likelihood (ML) method using the algorithm of Jukes–Cantor with 1000 bootstrap resampling of the data to test the tree topologies.

## Results

### Current Bacterial Wilt Occurrence in Central and Northern Peru

Samples were collected from the central and northern departments of Peru, Piura, Amazonas, Ancash, Cajamarca, and Huanuco, based on consultation with the local phytosanitary organization SENASA and agricultural innovation center INIA. The specialists identified the locations of potato fields or other Solanaceous crops with symptoms similar to bacterial wilt infection. The approximate field size varied from 0.1 to 3 ha, destined either for own consumption or for selling and the seed was either self-produced or commercially purchased. Infected samples came from both types of fields, confirming that although diagnostic tests for *R. solanacearum* are available in Peru (Priou et al., 1999, 2010) formal disease diagnostics is not common practice before seed trading. The most popular cultivars were Yungay and Amarilis, followed by Unica, Perricholi, Capiro, Chaucha Amarilla, and Huayro Blanco (**Table 1**).

**Table 1:** Details of the fields or water ways sampled in the departments of Peru during 2016, and number of infected samples detected.

Field number	Altitude m.a.s.l.	Area ha	Potato cultivar	Number of stem samples collected (positive samples)	Number of water samples collected
**Amazonas**					
1	2672	0.04	Amarilla	26 (24)	0
2	2915	0.2	Huayro blanco	16 (2)	0
3	2697	0.2	Amarilis	1 (0)	0
4	2824	0.2	Canchan	3 (0)	0
			**Total**	**46 (26)**	**0**
**Ancash**					
1	3154	0.06	Yungay	25 (0)	1 (1)
2	3406	0.08	Yungay	6 (6)	1 (0)
3	3656	0.12	Yungay	4 (0)	0
4	3628	0.06	Mixture of native varieties	2 (0)	1 (0)
5	3742	0.04	Mixture of native varieties	1 (0)	0
6	3321	0.1	Yungay	2 (0)	0
7	3327	0.08	Yungay	2 (0)	1 (0)
8	3095	0.05	Yungay	2 (0)	1 (0)
			**Total**	**44 (6)**	**5 (1)**
**Cajamarca**					
1	3002	0.5	Amarilis	2 (0)	0
2	3381	1	Yungay	1 (0)	0
3	2781	0.1	unknown	4 (4)	0
4	2312	0.6	Amarilis	1 (0)	0
5	2111	0.1	Unica	10 (10)	0
6	3680	0.5	Yungay	1 (0)	0
7	2159	0.3	Amarilis, Perricholi, Chaucha Amarilla	12 (12)	0
8	2193	na	^∗^sampled from river	0	1 (0)
9	2779	0.5	Amarilis, Yungay	10 (10)	0
10	2748	0.25	Chaucha amarilla	15 (15)	0
11	2635	0.3	Perricholi	2 (2)	0
12	2806	1	Amarilis, Unica	12 (12)	0
13	2407	1	Unica, Amarilis, Perricholi	9 (0)	0
14	2386	1	unknown	14 (0)	0
15	2216	1	unknown	18 (1)	0
16	2084	1	Amarilis	14 (0)	0
17	2075	1	Amarilis, Unica	20 (0)	0
18	2441	1	Unica	6 (0)	0
19	2761	1	Unica, Amarilis	16 (1)	0
			**Total**	**167 (67)**	**1 (0)**
**Huanuco**					
1	2596	1	Yungay	9 (7)	1 (1)
2	3350	1.5	Capiro	15 (7)	0
3	3350	0.5	Capiro	15 (4)	0
4	3350	1	Capiro	15 (11)	0
			**Total**	**54 (29)**	**1 (1)**
**Piura**					
1	2847	0.1	Huayro blanco	7 (7)	0
2	2759	0.2	Amarilis	6 (0)	0
3	2888	0.1	Amarilis	19 (17)	0
4	2911	2	Amarilis	4 (1)	0
5	2941	3	Amarilis	11 (0)	0
			**Total**	**47 (25)**	**0**


*Stem samples were collected from potato and pepper plants with wilting symptoms, and water samples were taken from the irrigation water or near-by river. Positive samples were identified by detecting Ralstonia solanacearum using Kelman method (Kelman, 1953)*.

The sampling focus was in potato crop, but if the farmers had other crops with wilting symptoms those were also collected. In total samples were collected from 38 potato fields and one pepper field. Of these 19, including the pepper field, were found infected with *R. solanacearum* (**Table 1**). The highest proportion of infections was found in Huánuco, where all fields with wilted samples had infected plants. In Ancash, *R. solanacearum* was found in the water sample collected from the irrigation canal of one of the fields, but nevertheless the wilted samples from this field were not infected. Only one of the fields sampled in Ancash was infected. Two separate prospections were made in Cajamarca, the first one in March and the second one in October 2016 and out of the 18 fields sampled 8 were found with infected plants. In Piura four of the five fields we visited had infected samples. In Amazonas, we visited four fields and two of them had infected samples. In many cases the wilting observed was apparently caused by other pathogens since of the 358 wilted samples collected, only 43% were found to contain *R. solanacearum*. The altitude of the sampling sites ranged from 2111 to 3742 m.a.s.l. and *R. solanacearum* was found in samples up to the altitude of 3406 m.a.s.l (**Table 1**).

### Molecular and Biochemical Characterization

The 265 *R. solanacearum* strains from Peru characterized in this study have been collected in Peru between the years 1966 and 2016 mostly from potato, but also from pepper, tomato, plantain, or soil (Supplementary Table S1). Based on the partial sequence of the endoglucanase gene, all new isolates collected at 2016 and all but one old isolate, were Phylotype II. One single isolate collected from tomato at the 1960’s turned out to be phylotype I.

**FIGURE 1:**
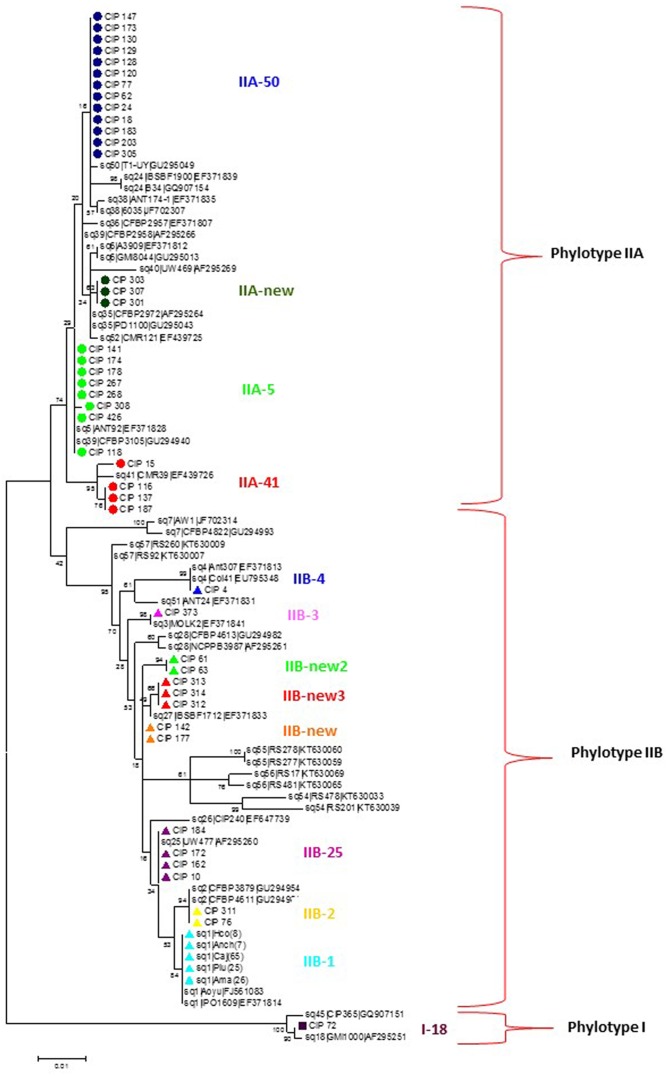
Molecular Phylogenetic analysis of the partial endoglucanase gene sequences (622 nt) of 90 *Ralstonia solanacearum* isolates from Peru and reference isolate sequences obtained from NCBI sequence database. The phylogeny was constructed using the Maximum Likelihood method. The tree with the highest log likelihood (–2108.4692) is shown. The percentage of trees in which the associated taxa clustered together is shown next to the branches. Initial tree(s) for the heuristic search were obtained automatically by applying Neighbor-Join and BioNJ algorithms to a matrix of pairwise distances estimated using the Maximum Composite Likelihood (MCL) approach, and then selecting the topology with superior log likelihood value. The tree is drawn to scale, with branch lengths measured in the number of substitutions per site. Circles, triangles and squares indicate sequences of phylotypes IIA, IIB and I, respectively, determined in this study, colors indicate different sequevars. Evolutionary analyses were conducted in MEGA7 (Kumar et al., 2016).

Phylogenetic analysis divided the Peruvian *R. solanacearum* isolates into three main clusters following the Phylotype classification (**Figure 1** and **Table 2**). The Phylotype IIB isolates formed seven sequevar groups including the previously identified sequevars 1, 2, 3, and 4 (Poussier et al., 2000; Wicker et al., 2007; Cellier and Prior, 2010). CIP-184, CIP-172, CIP-162, and CIP-10 share similarity to sequevar 25 (UW477, Poussier et al., 2000), but the grouping does not receive strong bootstrap support. Nevertheless, these isolates are here considered belonging to sequevar 25 (**Figure 1** and **Table 2**). In fact, CIP10 has been sequenced before and is classified as sequevar 25 (Poussier et al., 2000). Some Peruvian isolates that group together with the reference sequevars previously assigned as Phylotype IIB strains IIB-57, IIB-51, IIB-28, IIB-26, IIB-27, IIB-55, IIB-56, IIB-54 (Poussier et al., 2000; Wicker et al., 2007; Cellier and Prior, 2010; Santiago et al., 2016) cluster in sub-groups supported by high bootstrap values in the phylogenetic analysis and are thus considered as new sequevars (**Figure 1**). Isolates CIP-61 and CIP-63 form a new sequevar of their own (IIB-new2), while CIP-312, CIP-313, CIP-314 belong to another new sequevar (IIB-new3). Phylotype IIA isolates from Peru clustered together with reference strains previously assigned to sequevars IIA-5, IIA-39, IIA-41, and IIA-50 (Wicker et al., 2007: Toukam et al., 2009; Cellier and Prior, 2010). Isolates CIP-301, CIP-303 and CIP-307 are classified as a new sequevar (IIA-new) since they are separated from the other previously identified IIA reference sequevars IIA-38, IIA-24, IIA-36, IIA-6, IIA-35, IIA-52 (Wicker et al., 2007, 2012; Toukam et al., 2009; Cellier and Prior, 2010; Lebeau et al., 2011; Hong et al., 2012) included in the phylogenetic analysis (**Figure 1**). The single Phylotype I isolate, CIP-072, was found in the banks of Amazon river in Loreto and had been determined as a biovar 3 (Martin et al., 1981). It shares similarity to sequevar 18 strain GM1000 isolated from tomato in French Quiana (Cellier and Prior, 2010).

**Table 2:** Endoglucanase (*egl*) sequevars identified among the sample of 265 *R. solanacearum* isolates collected from different hosts, soil, or water in Peru 1966–2016.

Phylotype-sequevar	Number of isolates	Host plant	Year	Reference strain
I-18	1	Tomato	1966	GMI1000| AF295251


IIA-41	4	Potato	1973-1981	CMR39| EF439726


IIA-5	8	Potato (7), Tomato (1)	1978-1991	ANT92| EF371828


IIA-50	13	Potato	1975-1988	T1-UY| GU295049


IIA-new	3	Potato	1988	Na


IIB-1	221	Pepper (6), Potato (207), soil (1), tomato (5), water (2)	1973-2016	IPO1609| EF371814.1


IIB-2	2	Potato	1976, 1988	CFBP4611| GU294981 CFBP3879| GU294954


IIB-25	4	Potato (3), soil (1)	1979, 1980	UW477| AF295260


IIB-3	1	Tomato	1989	MOLK2| EF371841


IIB-4	1	Plantain	1966	Ant307| EF371813 Col41| EU795348


IIB-new	2	Potato	1978, 1979	Na


IIB-new2	2	Potato	1976	Na


IIB-new3	3	Eggplant (2), Tomato (1)	1989	Na


Total	265			




*Na, Not available*.

**FIGURE 2:**
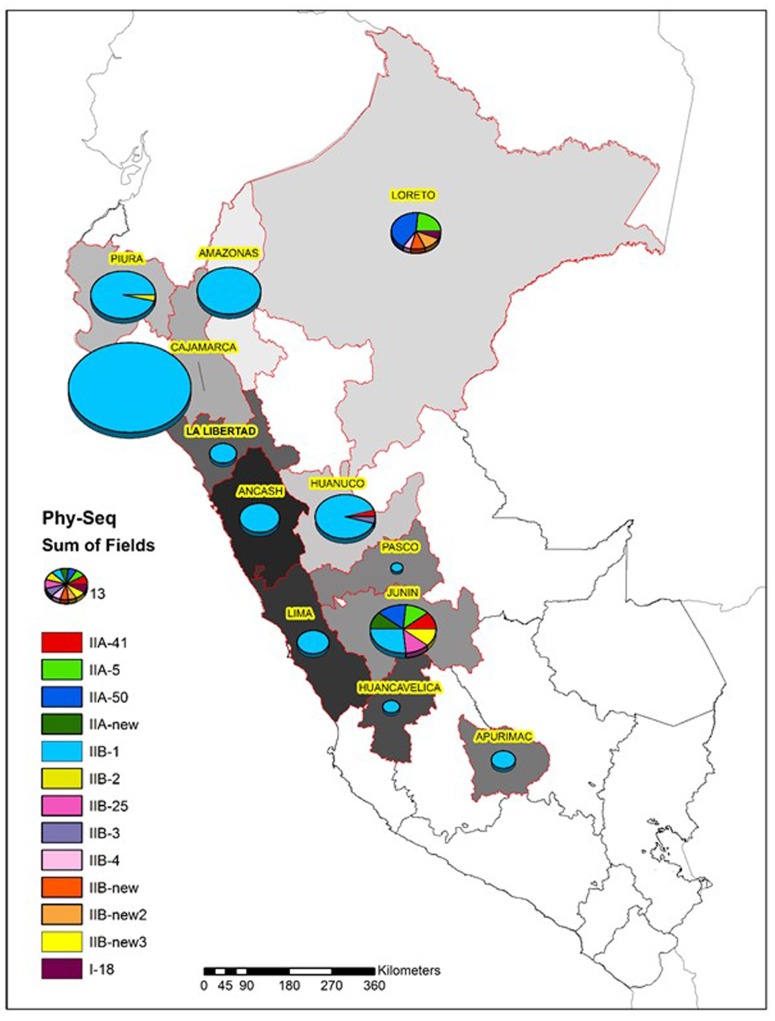
Diversity of the *R. solanacearum* isolates collected in Peru 1966–2016 based on the phylotype and the partial sequence of the endoglucanase gene (*egl*) (Fegan and Prior, 2005). The size of the pie is proportional to the number of isolates analyzed from each Peruvian department.

As expected, most of the Peruvian *R. solanacearum* isolates were IIB-1 strains. In the old collection sampled between 1966 and 2013, 72% (92/121) were IIB-1 and in the new collection at 2016 no other strains were found. Since all isolates analyzed at 2016 had identical *egl* sequences only five were left in the final phylogenetic tree to represent each of the Peruvian departments Huanuco (Hco), Ancash (Anch), Cajamarca (Caj), Piura (Piu), and Amazonas (Ama) that were sampled. Looking at the geographical differentiation in terms of sequevar diversity, departments of Junin with seven and Loreto with five different sequevar groups, contained the greatest diversity (**Figure 2**). Unfortunately, we were unable to obtain samples from these locations at 2016 and thus cannot confirm the level of current diversity at these locations.

All Peruvian Phylotype IIA isolates had the biovar 1 phenotype while the Phylotype IIB isolates were more variable in this respect consisting of isolates belonging to biovar 2A, 2T or 1 phenotypes (**Table 3**).

**Table 3:** Phylotype, *egl* sequevar and biovar of the 265 *R. solanacearum* isolates from Peru.

Phylotype-sequevar	Biovar			
	1	3	2A	2T
I-18	–	1	–	–
IIA-41	4	–	–	–
IIA-5	8	–	–	–
IIA-50	13	–	–	–
IIA-new	3	–	–	–
IIB-1	5	–	216	–
IIB-2	–	–	2	–
IIB-25	–	–	–	4
IIB-3	1	–	–	–
IIB-4	1	–	–	–
IIB-new	–	–	–	2
IIB-new2	–	–	–	2
IIB-new3	–	–	–	3
Total	35	1	218	11


### Pathogenicity

**FIGURE 3:**
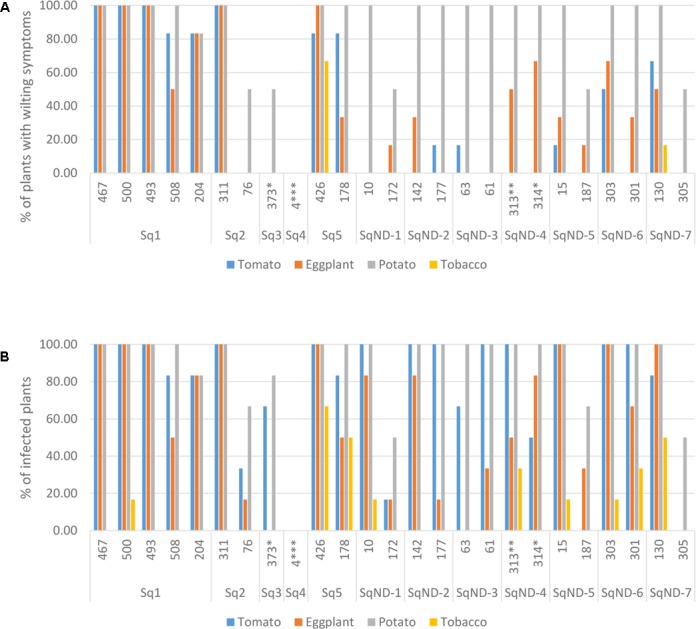
Pathogenicity of 25 *R. solanacearum* isolates on tomato, eggplant, potato, and tobacco measured as proportion of wilted plants **(A)** and proportion of latent infections by MKM test **(B)**. The ID number and the *egl* sequevar group of each isolate are indicated on the x-axis. ^∗^isolated from tomato, ^∗∗^isolated from eggplant, ^∗∗∗^isolated from plantain.

We selected 25 isolates representing the different *egl* sequevar groups (**Figure 1**) for pathogenicity tests using pepper, potato, tomato and tobacco. Most of these isolates originate from potato, but two are from tomato, one from eggplant and one from plantain (**Figure 3**). All isolates, except for the one originating from plantain, were pathogenic on at least one of the tested plants. The plantain isolate may have lost its pathogenicity during storage, but we cannot be sure since we did not attempt to inoculate its original host. Potato was susceptible to all the other isolates, resulting in wilting symptoms in 50–100% of the plants. Almost all isolates caused latent infection in tomato and eggplant, but wilting was observed much less frequently. Tobacco was least affected by the isolates tested as most of the isolates were nonpathogenic to this plant. Only two isolates belonging the phylotype IIA (CIP-426 and CIP-130) caused wilting in all hosts tested. The phylotype IIA seems more adapted to infect tobacco than the phylotype IIB strains as nearly all IIA isolates caused latent infection in this plant. IIB-1 and IIB-2 isolates were very aggressive on potato, tomato and eggplant, but all other IIB sequevars, except for IIB-4, caused wilting only on potato. Thus, these other IIB sequevars may have different pathogenicity mechanisms as they do not cause wilting in tomato but result in only latent infection.

## Discussion

This study describes the current bacterial wilt infestation level in the potato fields in the Peruvian Andes and is the first extensive molecular characterization of the bacterial wilt pathogen *R. solanacearum* in Peru. To investigate the occurrence of wilting due to this pathogen and to unravel its genetic diversity we embarked on several prospections that included the main potato growing areas in the central and northern parts of the country in the slopes of the Andes mountains. We sampled in total 39 farmers’ fields located between the latitudes of S05°09′53.0″ and S10°16′19.1″ and altitudes 2111–3742 m.a.s.l. where wilting symptoms had been reported by farmers or phytosanitary officials. Symptoms in the fields included wilting, in some cases of one branch in the hill only, a symptom that is very typical for *R. solanacearum* infection. In some cases, after removing the plants from the soil, the underground part of the stem was found damaged, either by insects or other pathogens. While symptomatic plants were found in almost all the fields visited, the frequency of *R. solanacearum* detected on potato was relatively low in the areas sampled during 2016. Although fields were pre-selected based on reported wilting symptoms, in less than 50% of the fields we found plants infected by *R. solanacearum* suggesting that other pathogens are in many cases responsible for the wilting symptoms observed. This means that the presence of the *R. solanacearum* in the field cannot be reliably determined only based on symptoms, but needs to be verified by a detection method such as NCM ELISA or PCR using specific primers. We used the Kelman’s cultivation method (Kelman, 1953) to detect the bacterium, which should be sensitive enough to enable the detection also of latent infections since it has been successfully used to confirm positive NCM ELISA tests from un-symptomatic samples (Priou et al., 1999, 2000, 2010). Taking a representative tuber sample after the harvest is recommended to catch the symptomless latent infections. However, since our prospections were made during the flowering period, we focused on sampling only stems. The previous prospections were made in the 1990s when seed lots from symptomless potato crops from Peru and Bolivia were tested for latent infection by *R. solanacearum* (Priou et al., 1999, 2010). In that study, tuber lots and stems from sixteen fields in Cajamarca and La Libertad departments exhibiting either no symptoms or low incidence of wilted plants (0.1–3%) were sampled and analyzed for presence of *R. solanacearum* by NCM ELISA test. All seed lots from the fields with low incidence of bacterial wilt were found positive for *R. solanacearum*, and roughly 50% of the seed lots coming from the apparently healthy fields were also positive (Priou et al., 1999). Cajamarca was also sampled in our study, where we collected wilted samples from 19 fields and found *R. solanacearum* only from nine of them suggesting that at least in Cajamarca the overall incidence of brown rot is now much less than it used to be. Some of this difference could be explained by the different detection methods and sampling units (stem vs tuber) used between the two studies, but most likely the reduction has taken place because of the intensive farmer training conducted by SENASA and CIP at 2003–2005 in the integrated management of bacterial wilt in potato (Anonymous, 2005). While this is a positive development, the disease is far from being eradicated and if the use of uncertified seed in potato production remains common practice, brown rot will also continue to be present. Only extremely strict hygiene measures in seed production and separating commercial seed from the ware potatoes can lead to a successful eradication of this bacterium (French, 1994; Janse, 2012). Most of the fields sampled were rather small plots (less than 1ha) and the smallest ones were used for family’s own consumption rather than selling in the market, which may affect the choice of tuber seed source and other cultivation methods. Our research confirms again that the bacterial wilt pathogen can survive in high altitudes as we found infected samples from the fields located above 3000 m.a.s.l. This has been reported before in the Andes of Peru and Bolivia (French, 1986; Castillo and Plata, 2016). The higher altitudes in the Andes are often used as potato seed production areas because these generally have lower disease incidence. The ability of brown rot to survive in the high altitudes means that there are less and less areas available for clean seed production by farmer’s conventional means.

We analyzed the genetic diversity and phylogeny of the *R. solanacearum* isolates collected in Peru from potato, tomato, pepper, plantain, soil, and water between 1966 and 2016. As expected all isolates, with one exception, belonged to the phylotype II supporting the association between phylotype and geographic region of origin (South America). The departments and areas visited at 2016 included many of those that were also represented in the historical collection and for all these the same IIB-1 strain was dominating. The Andes is thought to be the origin of the *R. solanacearum* phylotype IIB sequevars 1 and 2 (previously race3 biovar 2) causing potato brown rot (Champoiseau et al., 2009). This strain has also spread through the tropical highlands and subtropical warm-temperate areas throughout the world, and is considered a quarantine pathogen in Europe (Janse, 2012) and a select agent in the United States under the Agricultural Bioterrorism Protection Act (Hawks, 2002). The IIB-1 strain is considered cold-tolerant as it can cause disease in cool conditions, but has a relatively narrow host range (Milling et al., 2009; Cellier and Prior, 2010). In our pathogenicity tests the IIB-1 and IIB-2 strains, the biovar 2T strains IIB-25, IIB-new and all IIA strains were highly aggressive on potato and causing latent infection also in tomato and eggplant. There was a clear difference in pathogenicity on tobacco between the two phylotypes IIA and IIB. All IIA strains were causing at least latent infection on tobacco and the strains IIA-5 and IIA-50 also caused wilting of this species, while IIB strains caused only few latent infections. IIB-3 isolate CIP-373 originating from tomato caused wilting in potato but in tomato only led to latent infection, while IIB-4 isolate CIP-4 originating from plantain was found nonvirulent. Previous research has shown that IIB-3 and IIB-4 strains are generally virulent to susceptible potato and tomato varieties (Cellier and Prior, 2010). The reduced virulence of some isolates could be attributed to the long time they have spent in the storage.

Evidently IIB-1 is currently the dominating *R. solanacearum* strain infecting potato in Peru and it is not known whether the other phylogenetically distinct strains still exist. Two isolates collected in Piura and Cajamarca at 1982 and 1976, respectively, grouped together with IIB-2 reference strains (Cellier and Prior, 2010) forming a phylogenetically separated group from the IIB-1 strains (**Figure 1**). This separation has been noted before for potato and plantain infecting strains from Colombia by microarrays (Cellier et al., 2012). The IIB-25, IIB-new, IIA-5, IIA-50, IIA-41, IIA-new, and I-18 were originally discovered in lowland areas where potato is normally not cultivated, because of the too warm climate for this crop. Research in 1970s and 1980s was conducted in these locations to study potato heat tolerance and the *R. solanacearum* strains were assumed endemic to the area (Martin et al., 1981). Since potato seed production usually takes place at higher altitudes with cooler climates the cold tolerant IIB-1 strain may have a better fitness and thus better chance for survival than the low land strains. The finding that the strains IIA-50 and IIA-41 that were also collected in Cajamarca and Huanuco, respectively, in the early 1980’s were no longer discovered in these areas in 2016 supports this idea.

The greatest diversity was discovered in the department of Junin in the warm jungle areas of San Ramon and in the department of Loreto at Yurimaguas, where some new sequevars of phylotype II and one isolate of phylotype I were found. Considering that these strains are probably endemic the high level of diversity encountered is expected and similar variability has been reported before in Brazil, especially for phylotype IIB by Santiago et al. (2016) who reported four new sequevars for this phylotype some of which were found on potato. Here we report additional three new sequevars of phylotype IIB that are phylogenetically distinct from any of those described before. The next prospections in Peru should focus on these diversity hotspots to check for the current occurrence of these strains. The old biovar 3 strain CIP-072 collected by French and Sequeira (1970) in the Amazon basin turned out to be phylotype I sequevar 18, which has been also found in other warmer climates in the Americas, such as Brazil (Santiago et al., 2016), Guatemala (Sanchez Perez et al., 2008), French Guiana (Deberdt et al., 2014), and Trinidad y Tobago (Ramsubhag et al., 2012). The I-18 strain has mostly been associated with tomato

## Conclusion

High molecular diversity was found among the historical collection of *R. solanacearum* strains sampled in Peru. However, current epidemics are caused by a clonal brown rot strain IIB-1. Nearly all isolates were highly aggressive on potato causing severe wilting and were also virulent on tomato, eggplant and tobacco to variable degrees. The most variable strains in the historical collection were found in areas that are no longer used for potato cultivation and thus these strains do not pose a real threat in the current landscape of potato production in the country. More discriminant molecular analysis using multilocus markers are under way to identify sources of contamination and patterns of distribution within the clonal dominating strain IIB-1.

## Author Contributions

LG: Collected samples from the field, conducted and supervised the laboratory work, conducted and supervised the pathogenicity assays, drafted and revised the manuscript. JH: Collected samples from the field, conducted laboratory work and pathogenicity assays, conducted sequence analysis, drafted the manuscript. EF: Collected samples from the field, conducted laboratory work and sequence analysis, revised the manuscript. JK: Conceived funding for the project, designed the work, supervised and conducted analysis of sequence data, did data interpretation, revised the manuscript. HL-K: Conceived funding for the project, designed the work, collected samples from the field, supervised and conducted analysis of sequence data, did data interpretation, drafted and revised the manuscript.

## Conflict of Interest Statement

The authors declare that the research was conducted in the absence of any commercial or financial relationships that could be construed as a potential conflict of interest.
